# Supplementary material for: The associations of palliative care experts regarding food refusal: A cross-sectional study with an open question evaluated by triangulation analysis
Source: PLoS One. 2020 Apr 9;15(4):e0231312. doi: 10.1371/journal.pone.0231312 (PMC7145006; doi:10.1371/journal.pone.0231312)
Supplement: Attachment — Submitted filename: Response to Reviewers.docx [file pone.0231312.s001.docx]

Response to Reviewers

| General notes and explanations | | |
| --- | --- | --- |
| Many thanks to everyone involved for this very friendly and appreciative approach in the review process.  To all a happy new year 2020. | | |
| After we created the revision, we gave the document to Language Editing. In order to be able to distinguish the revisions from each other, the document "Revised Mansucript with Track Changes" shows the author's changes with a yellow background, the agency's changes for the linguistic revision in revision mode. | | |
| As the line numbers in the two documents differ considerably, the information refers to the document "Revised Manuscript with Track Changes". | | |
| Comments | Line | Changes & Responses |
| Manuel Fernández-Alcántara, Ph.D. |  | Thank you very much for your feedback and the chance of a review process. We have taken the comments from you and the reviewers into account and made the changes to the manuscript accordingly. Thank you very much! The comments are listed below and are commented on the right. |
| Please correct your reference to "p=0.000" to "p<0.001" or as similarly appropriate, as p values cannot equal zero. | 601 | Changes have been made, see Table 4 and figure 1 |
| Please remove any abbreviations and symbols from your title in your manuscript and on the online submission form | 1-6 | amended |
| We suggest you thoroughly copyedit your manuscript for language usage, spelling, and grammar. If you do not know anyone who can help you do this, you may wish to consider employing a professional scientific editing service.  Please provide the name of the colleague or the details of the professional service that edited your manuscript |  | The document has been revised by American Journal Experts (AJE). The certificate will be uploaded separately. |
| 4. Please ensure authors names are not duplicated on the online submission form |  | The authors have been reviewed and changed. |
|  | | |
| Reviewer #1:  Only 17.5% of participants completed the study. This percentage may indicate the little consideration of a topic such as the refusal of food. This aspect should be well emphasized and discussed. It should be noted that the practical application of the results of this study must be confirmed by other studies, before being considered valid for influencing the guidelines. | 1278-1285 | Thank you for your feedback. We added this statement in the section “Strengths and limitations”. |
|  | | |
| Reviewer #2: It is an interesting and good article. The manuscript focuses on interesting and sensitive issue that has important ethical implications for medical practice. It is adequately written. |  | Thank you very much for your kind words and the many good hints for revising the manuscript. We have made the changes with pleasure and are grateful for your good eye, your constructive suggestions and your appreciative nature. |
| According the authors, the strength and the limitation of the study are methodological aspects. Authors say: “The limitation of the study is mainly due to a method that is still little used and therefore difficult to comprehend. For this reason, care was taken to describe the procedure as precisely as possible”. However, the exactly name of method used is omitted. I consider it would be important to justify the use of mixed methods and briefly explain their main characteristics. As the authors indicate, if the method used is difficult to comprehend, this would help to understand it.  The type of design used is also omitted. Indicating, explaining and justifying it would also help to understand the methodological questions of the study. In this way, limitation of the study could be minor. | 205-300 | The method part, especially design and data analysis, was revised and references were added. We hope that the additional explanations will lead to a comprehensible understanding. |
| In relation to these same methodological questions and the difficulty for their understanding (as it is indicated by the authors themselves), it would be necessary to explain what the colors of the cells in table 3 mean. In addition, it would be more advisable not to use colors as indicators of specific issues. (There may be many readers who prefer to print the article to read it and a color printer is not available for them.) | Table 3  Line 578 | The colors were indeed not helpful and were therefore deleted. |
| In the same way, table 4 could be ameliorated. It would be more appropriate to use asterisks (*) to indicate statistically significant differences (not the color red). Further, why the meaning of the color red is explained but not the meaning of the bold font, gray color or square brackets []? | Table 4  Line 601 | Table 4 was revised, colors were removed and asteriks were inserted and described accordingly. |
| It is possible to clarify why lines 195-196 indicate that the category end-of-life was not clearly associated with dying? (p <0.001) |  | It was a mistake that we gratefully acknowledged. We have changed the appropriate passages. |
| It would be necessary to indicate in the Discussion Section future lines of research that extend the objective of this study, for example: are there differences between the answers of the participants depending on their profession or their work experience in years? | 1284-5 | This point has been included in the chapter Strenghts and limitations. |
| Other minor aspects: The full title is not precise / accurate. It would better a title that specifies more the content of the manuscript. (It is possible because the journal allows more characters to be used in the title). Further, it would be advisable that full title and short title not be exactly the same. | 1-6 | Both titles have been revised |
| It is advised to improve the Introduction section. It is too short. The state of the art is scarcely presented. | 89-113 | Introduction has been edited |
| The idea transmitted on lines 180 and 181 is repeated on lines 184 and 185. |  | The repetition has been deleted, thank you for that hint. |
| After the title of table 4 appears: “Significance per cell”. This must be removed. They are words that are isolated. In addition, this is already indicated at the beginning of line 192. | Table 4 | “Significance per cell” has been removed. |
| References should be reviewed. The Vancouver style (style demanded by Plos One) has not been adequately used. |  | Sorry, how embarrassing and unnecessary. I changed the style from Numbered to Vancouver. |
| In table 4 “ : ” must be eliminated after Physical aspect. | Table 4 | Merci - “:” has been removed. |
